# 265. Common Organisms of Parotid abscess in Cambodian Children

**DOI:** 10.1093/ofid/ofad500.337

**Published:** 2023-11-27

**Authors:** Prak Farrilend

**Affiliations:** Angkor Hospital for Children, siem reap, Siem Reap, Cambodia

## Abstract

**Background:**

Our aim is to determine the most common pathogens to cause parotid abscess and some epidemiology on children under 15 years of age who were admitted at Angkor Hospital for Children and consider where the children lived in order to aid critical thinking for quick diagnosis and proper treatment

**Methods:**

Our study is retrospective study chart review of all the children who had parotid abscess that was confirmed during surgery. The 42 patients were all under 15 years of age and lived in Cambodia. The duration of the study was 5 years, from 01-Jan-2017 to 01-Jan-2022

**Results:**

Of the 42 patients with parotid abscess, the most common pathogen found was Burkholderia Pseudomallei in 76% (32 of cases), of which 60% (19 cases) affected the right side and 40% affected the left side (13 cases) and we less commonly saw bilaterally. Staphylococcus aureus was the second most common cause and found in 24% (9 cases) of which 77% (7 cases) were on left side and only 23% (2 cases) on the right side. Cheek swelling was a chief complaint in 99% of cases and fever was a presenting feature in 90% of cases. They are from multiple different provinces in Cambodia that lie around the *Tonle Sap River*, primarily Siem Reap 77% (33), then Kampong Thom 9.5% (4), Battambang 5% (2), Phnom Penh 2.3% (1), and Banteay Meanchey 2.3% (1).

**Conclusion:**

The most common cause of parotid abscess in children under 15 years of age in our study was Burkholderia Pseudomallei which is Gram negative rod shape, and mostly involved the right side and was not seen in bilaterally. The other pathogen is Staphylococcus Aureus which more commonly effected the left side. The children all live in provinces surrounding the Tonle Sap River region.

For the regional of their living were from different province and place in that we found from the surrounding the *Tonle Sap River*, Siem reap 77% (33), Kampong Thom 9.5% (4), Phnom Penh2.3% (1), Battambang 5% (2), Banteay Meanchey 2.3% (1).

**Disclosures:**

**All Authors**: No reported disclosures

